# Comparison of MRI head motion indicators in 40,969 subjects informs neuroimaging study design

**DOI:** 10.1038/s41598-024-79827-9

**Published:** 2024-11-27

**Authors:** Thomas Wei Jun Teo, Seyed Ehsan Saffari, Ling Ling Chan, Thomas Welton

**Affiliations:** 1 National Neuroscience Institute, 11 Jalan Tan Tock Seng, Singapore, 308433 Singapore; 2 Duke-NUS Medical School, Singapore, Singapore; 3 Diagnostic Radiology, Singapore General Hospital, Singapore, Singapore

**Keywords:** Neuroscience, Magnetic resonance imaging

## Abstract

Head motion during MRI compromises image quality for clinical assessments and research. Active motion reduction strategies are effective but rarely applied due to uncertainty in their value for a given study. The ability to anticipate motion based on group characteristics would aid effective neuroimaging study design. This study compared putative motion indicators for their association to fMRI head motion in a large UK Biobank cohort (n = 40,969, aged 54.9 ± 7.5 years, 53% male). Body Mass Index (BMI; β_adj_ = .050, p < .001) and ethnicity (β_adj_ = 0.068, p < 0.001) were the strongest indicators of head motion. A ten-point increase in BMI, which is the difference between “healthy” and “obese”, corresponded to a 51% increase in motion. Findings were similar in a subgroup with no lifetime diagnoses (n = 6858). Motion was not significantly increased in individuals with psychiatric disorders, musculoskeletal disorders, or diabetes. The hypertension subgroup exhibited significantly increased motion (p = 0.048). Cognitive task performance (t = 110.83, p < 0.001) and prior scan experience (t = 7.16, p < 0.001) were associated with increased head motion. Our results inform decision making for implementation of motion reduction strategies in MRI. BMI outweighs other motion indicators, while blood pressure, age, smoking and caffeine consumption are relatively less influential. Disease diagnosis alone is not a good indicator of MRI head motion.

## Introduction

Head motion during MRI scanning is a major problem for clinical assessment and research studies because it introduces artefacts that reduce the quality of imaging and diagnostics. The effects of even sub-millimetre head motion are large enough to compromise the results of neuroimaging studies, including both functional and structural connectomics^1–4^, with more profound effects occurring in short-range correlational functional connectivity estimates^2,5^. Head motion is often uncontrollable, occurring even when subjects are instructed to limit motion, such as that caused by both physiological (e.g., respiration and swallowing)^6,7^ and non-physiological effects (e.g., tics, fidgeting, head tremor)^8^.

Retrospective correction is commonly applied to structural and functional MRI (fMRI) data to reduce the effects of head motion^9^. However, this remains limited because, besides intra-slice and intra-voxel information being affected, commonly-applied computational methods for retrospective motion correction leave residual artefacts of their own^10–12^ and can elicit false “activations” in fMRI experiments^13^. fMRI head motion can also be addressed by active mitigation strategies, such as real-time visual or tactile feedback, sedation^14,15^, acclimation^16,17^, splitting the acquisition into multiple sessions, introducing breaks^18^, use of personalised head moulds^19^, or behavioural training^20^. Such active strategies limit the root-cause motion itself, and are successful in improving image quality and plausibly reducing the costs for fMRI acquisitions (reportedly > 50%^21^). Despite this, active motion correction is not widely applied. This may be related to the need for additional planning and resources or difficulty in predicting the effect of motion for a given cohort, thus forfeiting potential benefit.

Studies have found associations between fMRI head motion with anthropometric, demographic, clinical, neuropsychological, behavioural and genetic factors. Respiratory and cardiac pulsations are common contributors to MRI head motion and elevated heart rate is associated with high blood pressure; thus, hypertensive patients generally have higher rates of cardiac pulsation and MRI head motion^22^. Diabetes can directly increase resting heart rates through hyperinsulinemia and elevated blood glucose levels^23–25^. Additionally, autonomic nervous system dysfunction is a common complication of diabetes, potentially affecting resting heart and respiration^26^. Overwhelming evidence supports the detrimental effects of smoking and its contribution to adverse cardiovascular events, chief among them being hypertension^27,28^. Smoking also contributes to the development of respiratory diseases that may encourage coughing^29^ and may increase general motion through behaviours such as fidgeting^30^. Reportedly, a substantial portion (8–40%) of the variance in head motion may be explained by a participant’s body-mass index (BMI)^6,31^. Obesity, a complex disease, is commonly defined by having a BMI of 30 kg/m^2^ or higher^32^, however there exists a proportion of obese individuals with high BMIs without the corresponding adiposity and cardiometabolic abnormalities, termed “metabolically healthy obese” (MHO) individuals^33^. Evidence has linked weight loss to systematic decreases in head motion during MRI^34^. Participants with greater BMIs could experience greater discomfort within the scanner, increasing respiratory effort and thus head motion^31^. The effects of age on motion have mostly been documented in younger populations, with increasing age inversely correlated with motion from toddlers up to young adolescents^35,36^. Patient demographics are often expected to exhibit greater motion; for example, those with disease^37^, psychiatric disorders^38^, ADHD^39^ or musculoskeletal disorders^40^.

There have been no large fMRI studies (n > 1000) on head motion across diseases; comparing subject indicators of head motion and quantifying their effects. Knowledge of the relative importance of subject indicators of MRI head motion could help in designing fMRI protocols for clinical trials and research studies, allow investigators to predict the amount of motion and potential value of active or retrospective strategies to address motion for a given cohort. Finally, it may be useful to estimate the effect that motion may have had on the results of previous studies.

The primary aim of our study is the comparison of MRI head motion indicators without initial selection of specific features. Our secondary aims are to (i) compare head motion across broad categories of disease, (ii) assess effects of cognitive task performance and task engagement on motion, and (iii) investigate effect of familiarity with MRI procedure on motion.

## Methods

### Study setting and selection of motion indicators

We used data from the UK Biobank (application number 82354): 502,461 MRI datasets were available from people aged 40–69 years. We selected putative indicators of fMRI head motion identified from the literature for evaluation^22–31,34–40^. The details of their measurement and format in the UK Biobank database are listed in Table 1.

**Table 1:** Putative indicators of fMRI head motion within the UK Biobank.

Indicator	UK Biobank Field ID	UK Biobank Field Name	Recoded data format
Diabetes	2443	Diabetes diagnosis	0 = No Diabetes 1 = Diagnosed
Sex	31	Sex	0 = Female 1 = Male
Education	6138	Qualifications	0 = A levels and below 1 = Bachelors and above
BMI	23,104	Body Mass Index	–
Time of Scan	21,862	Brain MRI sign-off timestamp	0 = AM 1 = PM
Past Smoking History	1249	Past tobacco smoking	0 = Never smoked 1 = Smoked previously
Ethnicity	21,000	Ethnic background	0 = British White 1 = Other ethnicities
Age	21,003	Age when attended assessment canter	–
Number of ICD10 Diagnoses	41,270	ICD10 Diagnoses	–
Mean Arterial Pressure*	4080 4079	Systolic Blood Pressure Diastolic Blood Pressure	–
Caffeine Consumption	3089	Caffeine drink within last hour	0 = No 1 = Yes

*Calculated from systolic and diastolic blood pressures recorded in UK Biobank using the formula, MAP = (SBP + 2 * DBP)/3.

### Data acquisition and filtering

UK Biobank resting-state and task-fMRI scans had the following parameters: GE-EPI, voxel-size = 2.4 × 2.4 × 2.4 mm, FOV = 88 × 88 × 64, TR = 0.735s, TE = 39 ms, multi-slice acceleration = 8x, FA = 52°, 490 timepoints for resting state (6 min) and 332 timepoints for task (4 min)^41^. Subjects included in the imaging arm were originally selected for safety and tolerability (e.g. did not have metal implants or certain surgeries, and were not claustrophobic)^42^. From amongst the entire database (n = 502,461), we first selected only subjects with complete baseline fMRI head motion data. For comparison, subjects were further filtered based on availability of the baseline and follow-up fMRI head motion data for resting-state fMRI and task fMRI scans. We then only included subjects with the relevant selected motion indicators available (Table 2). The final cohort included n = 40,969 subjects. Where possible, data fields with discrete data were recoded into binary variables for our analyses. Patients with recorded ICD10 diagnoses (UK Biobank field ID: 41,270) were grouped into four respective disease categories of interest and sufficient size (n > 500; Table S1).

**Table 2:** Sample characteristics.

Characteristic	Descriptive statistics
Overall sample, n	40,969
Subgroups	
Psychiatric Disorders, n	973 (2.39%)
Musculoskeletal Disorders, n	1095 (2.67%)
Hypertension, n	1232 (3.00%)
Diabetic, n	697 (1.7%)
Control, n	6858 (16.7%)
Age, years	54.9 ± 7.5
Body-mass index	26.5 ± 4.2
Mean Arterial Pressure	99.8 ± 12.2
Total number of ICD10 Diagnoses	7.3 ± 8.1
Ethnicity	White-British: 39,645 (97.6%) Other: 992 (2.4%)
Sex	Female: 21,711 (53.0%) Male: 19,258 (47.0%)
Education	“A” Levels and below: 18,835 (49.9%) Bachelors and above: 18,940 (50.1%)
Smoking	Never Smoked: 17,561 (42.7%) Smoked Previously: 21,717 (53.3%)
Caffeine Consumption 1h prior to scan	Yes: 793 (2.1%) No: 33,768 (97.9%)
Diabetes	Diabetic: 1,029 (2.5%) Non-Diabetic: 39,882 (97.5%)
Baseline resting-state fMRI head motion, mm	0.123 ± 0.06
Follow-up resting-state fMRI head motion, mm	0.121 ± 0.06
Baseline task fMRI head motion, mm	0.147 ± 0.06
Follow-up task fMRI head motion, mm	0.145 ± 0.06

### Quantification of MRI head motion

MRI head motion was previously quantified (in millimetres) for fMRI scans using FMRIB Software Library’s MCFLIRT, according to the UK Biobank Brain Imaging Documentation^41^. In short, the mean displacement across the whole brain was estimated for each consecutive pair of timepoints, and then averaged across all timepoints. For resting-state fMRI scans, this was encoded in UK Biobank field ID 25741 and, for task fMRI scans, in UK Biobank field ID 25742, with each having a separate instance for the baseline and follow-up MRI scan (instances 2 and 3 respectively).

### Planned statistical analysis

Statistical analysis was conducted using the SPSS software (Statistical Package for the Social Sciences (SPSS) software, version 26 IBM Corp., Armonk, NY, USA). Subjects’ characteristics were reported as mean and standard deviation for continuous variables and frequency and percent for categorical variables. Natural-logarithm transformation was performed on the motion data due to right skewed distributions. Univariate linear regression analysis was performed to examine the association of potential indicators as eleven independent variables and fMRI head motion as outcome variable. Normality assumption was assessed visually using Quantile–Quantile (QQ) plots of the residuals. Back-transformation was applied to the regression beta coefficients ($\beta$) for easier interpretation using the following formula, where ${\%}\,{\text{change in motion}}$ represents the percentage change in outcome variable (fMRI head motion) per one-unit change in the independent variable:

$${\%}\, {\text{change in motion}}={100(e^{\beta }-1)}.$$

Multivariable linear regression analysis was performed to adjust the association effect sizes for the potential confounders. All eleven variables were included in the multivariable model as they are found to be statistically relevant confounders. Multi-collinearity among the eleven variable was assessed via variance inflation factor analysis and no collinearity was found.

To compare MRI head motion across controls and disease categories, multivariable linear regression analysis was performed with the disease categories as the between-subjects factor, MRI head motion as the dependent variable, and the identified significant MRI head motion indicators from the above regression analyses included as covariates, adjusted for significant confounder variables identified in prior analyses. We did not apply multiple comparison corrections due to the small number of planned a priori tests, single outcome, and the non-exploratory nature of our analyses.

To test the difference in motion of fMRI scans performed at rest and during performance of a cognitive task, paired t-test was performed using the log-transformed fMRI head motion at rest and during task, and the normality assumption was assessed via QQ plot.

To examine the association between resting-state network BOLD activation during task performance and log task fMRI motion, accounting for statistically and clinically potential confounding indicators identified in prior analyses, multivariable linear regression was performed. The first test included subjects who had BOLD activation data and fMRI head motion data (n = 35,413), while the regression analysis included subjects that had BOLD activation data and task fMRI head motion data (n = 29,188). BOLD activation data were obtained from fMRI data involving face-shapes contrast tasks to reduce the amount of total movement available in other tasks requiring action (i.e. button pressing).

To compare motion between baseline and follow-up rest and task fMRI motion while accounting for exposure effects in the interim, we calculated the difference in motion between follow-up and baseline scans, and performed one-sample t-test. This test included subjects who had fMRI head motion data for both baseline and follow-up resting-state (n = 3240) and task fMRI (n = 2561) respectively.

The original statistical analysis plan was followed without any deviations during the data analysis process. No post-hoc or exploratory analyses were conducted.

## Results

### Sample characteristics

The included sample comprised 40,969 middle-to-older aged adults (54.9 ± 7.5 years). The sample was mostly White-British (97.6%), had slightly more females (53.0%) than males, and roughly half (50.1%) held a bachelor’s degree or higher. They were slightly overweight on average (BMI 26.5 ± 4.2), with 1.7% being diabetic (Table 2).

### Indicators of MRI head motion

In the univariate linear regression model (Table 3), diabetes (β = 0.289, ${\%}\,{\text{change in motion}}$ = 33.4%, p < 0.01) and male sex (β = 0.115, ${\%}\,{\text{change in motion}}$= 12.2%, p < 0.01) were the strongest indicators of higher fMRI head motion. All indicators were significantly associated with motion, except for caffeine consumption.

**Table 3:** Association of baseline patient characteristics with MRI head motion: univariate analysis.

Variable	Unit OR Category	Unadjusted Beta Coefficient (95% CI)	% Change in head motion*	P value	R^2^
Diabetes	0: No Diabetes 1: Diabetes	0.2885 (0.2757, 0.3014)	33.449	**0.0040**	0.012
Sex	0: Female 1: Male	0.1151 (0.1111, 0.1191)	12.200	** < 0.0001**	0.020
Education	0: “A” Levels and Below 1: Bachelors and above	-0.0973 (-0.0932, -0.1015)	-9.274	** < 0.0001**	0.014
BMI	kg/m2	0.0539 (0.0535, 0.0543)	5.535	** < 0.0001**	0.305
Time of Scan	0: Before 12PM 1: After 12PM	0.0539 (0.0495, 0.0582)	5.534	** < 0.0001**	0.004
Past Smoking History	0: Non-smoker 1: Smoker	0.0477 (0.0436, 0.0518)	4.885	** < 0.0001**	0.003
Ethnicity	0: British White 1: Other	0.0377 (0.0246, 0.0509)	3.847	** < 0.0001**	0.000
Age	Years	0.0101 (0.0099, 0.0101)	1.020	** < 0.0001**	0.035
Number of ICD10 Diagnoses	-	0.0096 (0.0094, 0.0099)	0.968	** < 0.0001**	0.036
Mean Arterial Pressure	mmHg	0.0077 (0.0075, 0.079)	0.772	** < 0.0001**	0.052
Caffeine Consumption	0: Non-drinker 1: Drinker	0.0064 (− 0.0082, 0.0210)	0.642	0.6621	< 0.001

Rows are ordered by the absolute unstandardised regression coefficient, from large to small. Variables in bold indicate p < 0.01, with effects on head motion represented as a percentage change per increase in variable unit. n = 40,969.

*Per unit change (continuous variables); in comparison to reference category (binary variables).

When accounting for intervariable effects between indicators (Table 4), the strongest indicators were instead non-White ethnicity (β_adj_ = 0.068, ${\%}\,{\text{change in motion}}$ = 7.08%, p < 0.01) and increased BMI (β_sadj_ = 0.050, ${\%}\,{\text{change in motion}}$= 5.13%, p < 0.01), while diabetes (β_adj_ = 0.115, ${\%}\,{\text{change in motion}}$ = 12.2%, p < 0.01) and male sex (β_adj_ = 0.044, ${\%}\,{\text{change in motion}}$= 4.47%, p < 0.01) were also still strong indicators. A single BMI-unit increase in BMI corresponded to a 5.1% increase in MRI head motion; accordingly, an obese individual would have 51% increased motion compared to a healthy-weight individual (a ten-point BMI difference). Each additional decade of age corresponded to a 7.2% increase in head motion.

**Table 4:** Multivariate association of baseline patient characteristics with MRI head motion.

Variable	Adjusted Beta Coefficient (95% CI)	% Change in head motion*	P value	η^2^	R^2^
Ethnicity (Others vs British White)	0.0684 (0.0567, 0.0801)	7.080	** < 0.0001**	< 0.001	< 0.001
BMI (kg/m^2^)	0.0501 (0.0496, 0.0505)	5.133	** < 0.0001**	0.233	0.269
Presence of Diabetes	0.0471 (0.0352, 0.0591)	4.827	** < 0.0001**	< 0.001	0.005
Male Sex	0.0438 (0.0400, 0.0475)	4.473	** < 0.0001**	0.003	0.009
Time of Scan (After 12PM)	0.0429 (0.0389, 0.0468)	4.380	** < 0.0001**	0.004	0.005
Education (Bachelor and Above)	− 0.0445 (− 0.0482, − 0.0409)	-4.355	** < 0.0001**	0.003	0.008
Caffeine Consumption	0.0216 (0.0079, 0.0353)	2.184	**0.1**16	< 0.001	< 0.001
Past Smoking History	0.0102 (0.0066, 0.0139)	1.028	**0.005**	< 0.001	0.001
Age (year)	0.0071 (0.0069, 0.0074)	0.715	** < 0.0001**	0.016	0.021
Number of ICD10 Diagnoses	0.0035 (0.0032, 0.0037)	0.348	** < 0.0001**	0.004	0.016
Mean Arterial Pressure (mmHg)	0.0016 (0.0014, 0.0017)	0.155	** < 0.0001**	0.002	0.025

Rows are ordered by the magnitude of their absolute unstandardised regression coefficient, from high to low. Variables in bold indicate p < 0.01, with effects on head motion represented as a percentage change per increase in variable unit. n = 40,969.

Eta-squared (η^2^) presents the corresponding effect size of explained variance; R-squared (R^2^) calculated using Lindeman, Merenda and Gold (LMG) method reflecting the explained variance.

*Per unit change (continuous variables); in comparison to reference category (binary variables).

In the multiple linear regression performed on a subset of participants with no recorded lifetime ICD10 inpatient diagnoses (Table 5), ethnicity (β_adj_ = 0.069, % change per unit = 7.14%, p < 0.01) and BMI (β_adj_ = 0.051, % change per unit = 5.24%, p < 0.01) were again the strongest indicators of MRI head motion. In contrast to the main dataset, past smoking history was not significantly correlated with motion.

**Table 5:** Results of the multiple linear regression to predict MRI head motion on a subset of 6858 participants with no recorded lifetime ICD10 inpatient diagnoses.

Variable	Adjusted Beta Coefficient (95% CI)	% Change in head motion*	P value	η^2^	R^2^
Ethnicity (Others vs British White)	0.0690 (0.0433, 0.0946)	7.140	**0.0072**	< 0.001	< 0.001
BMI (kg/m^2^)	0.0511 (0.0499, 0.0523)	5.243	** < 0.0001**	0.024	0.268
Male Sex	0.0363 (0.0275, 0.0451)	3.695	** < 0.0001**	0.002	0.009
Education (Bachelor and Above)	-0.0326 (-0.0413, -0.0240)	-3.212	** < 0.0001**	0.002	0.004
Time of Scan (After 12PM)	0.0303 (0.0212, 0.0394)	3.073	**0.0009**	0.003	0.003
Caffeine Consumption	0.0278 (-0.0031, 0.0588)	2.824	0.3680	< 0.001	< 0.001
Past Smoking History	0.0104 (0.0019, 0.0189)	1.044	0.2235	< 0.001	< 0.001
Age (year)	0.0061 (0.0055, 0.0067)	0.615	** < 0.0001**	0.012	0.015
Mean Arterial Pressure (mmHg)	0.0025 (0.0021, 0.0029)	0.248	** < 0.0001**	0.005	0.034

Rows are ordered by the adjusted regression coefficient, from high to low with effects on head motion represented as a percentage change per increase in variable unit. n = 6858.

*Per unit change (continuous variables); in comparison to reference category (binary variables).

### MRI head motion in disease

**Fig. 1:**
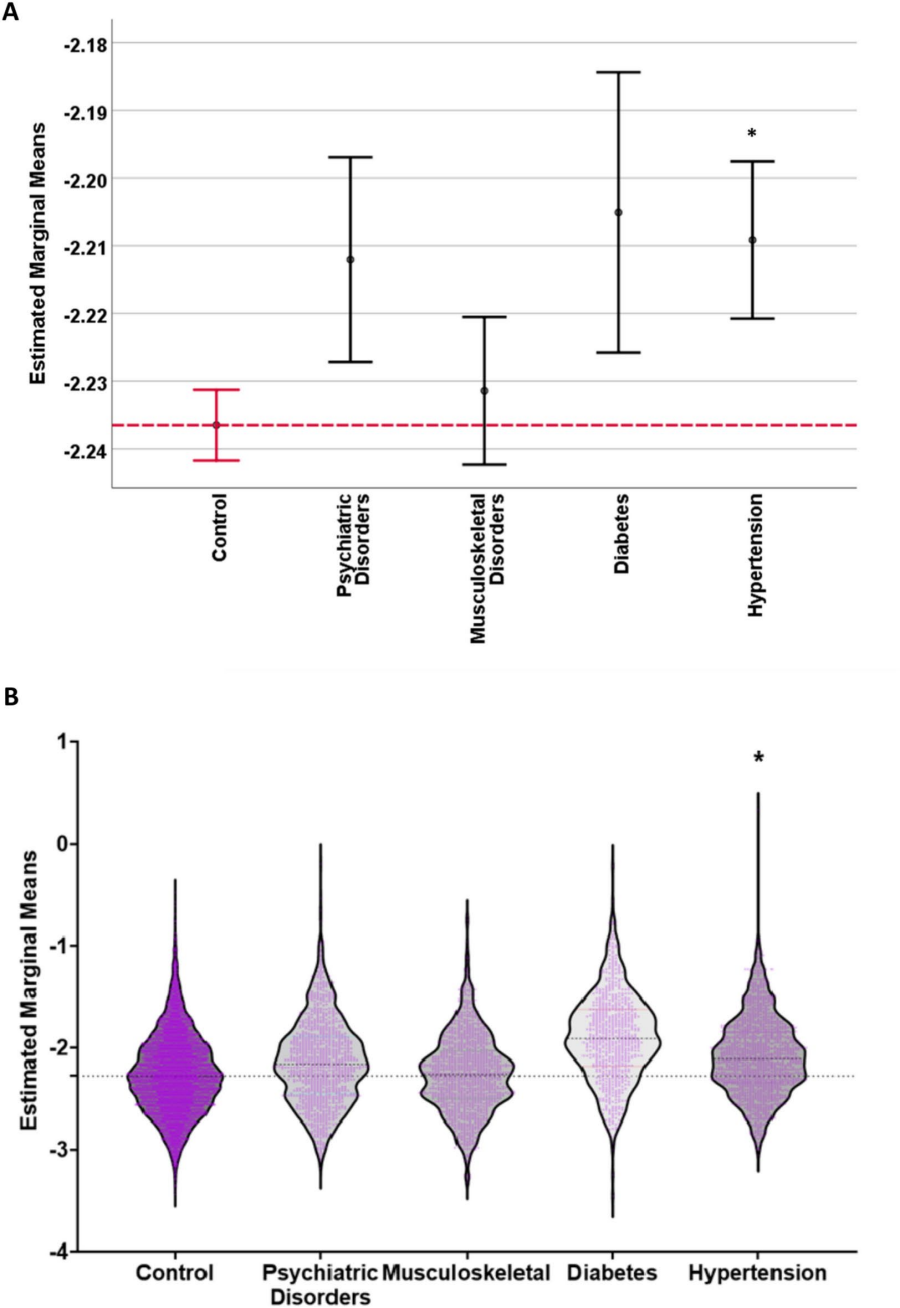
Effects of lifetime inpatient disease diagnoses, broadly defined, on log resting-state fMRI motion, accounting for effects of covariates. (**A**) Estimated marginal mean of control group log motion is denoted by the red reference line. Error bars denote the standard error. (**B**) Raincloud plot providing overview of effect sizes and variability. Both figures * = significantly different from controls.

One-way ANCOVA was conducted to compare the effects of lifetime inpatient disease diagnosis on fMRI head motion against controls while controlling for the indicators of MRI head motion we had analysed previously (Fig. 1). All disease groups exhibited increased motion relative to the control group. We found a significant difference from controls only for the hypertension group (mean difference = 0.0270, p < 0.05). (Table S2).

### Impact of performing an fMRI cognitive task on motion

Paired samples t-test to compare motion between non-motor cognitive task and resting-state fMRI head motion showed that mean fMRI head motion was 20.6% higher during cognitive task performance than at rest (mean difference = 0.0250, SD = 0.0425, t (35,412) = 110.83, p < 0.001, 95% CI [0.0246, 0.0255]).

Furthermore, higher log BOLD activation during task performance was significantly correlated with log task fMRI motion (β = 0.146, % change per unit = 15.7%, p < 0.001). When accounting for continuous MRI head motion indicators, the test also yielded similar results (β = 0.136, % change per unit = 14.6%, p < 0.001).

### Impact of prior scan experience on fMRI head motion

fMRI head motion was significantly higher during follow-up than baseline scans for both rest (mean difference = 0.005, SD = 0.042, t(3239) = 7.158, p < 0.001, 95% CI [0.0038, 0.0067]) and task fMRI (mean difference = 0.008, SD = 0.001, t(2560) = 8.448, p < 0.001, 95% CI [0.0061, 0.0099]) conditions.

## Discussion

Head motion presents a significant challenge to the acquisition and analysis of high-quality fMRI data for clinical and research assessments. We investigated the association between fMRI head motion and eleven putative indicators of head motion in the largest study to date by two orders of magnitude, and found that ethnicity and obesity far outweigh other indicators when appropriate adjustments are made. For obese individuals, fMRI motion is 51% greater than comparable healthy-weight individuals. These findings were consistent across individuals with or without lifetime inpatient hospital diagnoses. All investigated categories of disease exhibited increased motion compared to controls apart from musculoskeletal disorders (including atherosclerosis). Performance of a cognitive task during fMRI scanning increased motion by 20%, and familiarity with MRI scanning increased motion by 15%. Our data address common—but unsubstantiated—assumptions about motion in patient groups and under different scanning conditions. Our results facilitate evidence-based decisions about motion correction of fMRI data by allowing researchers to predict motion, and thus helping to inform the inclusion of active motion mitigation in study protocols.

We found that a 10-point increase in BMI was associated with a striking 51% increase in head motion, corresponding to the difference between healthy and obese weight. Our findings are consistent with those of previous imaging studies, where BMI was consistently identified as one of the most important indicators of head motion^6,31,34^ (accounting for 8–40% of variance in head motion, versus 5% in our study). Sources of uncontrollable head motion include respiration and cardiac pulsation, thus, it is unsurprising that higher BMI increases head motion, given that increases in BMI generally lead to higher heart and respiratory rates^43–45^. Moreover, higher-BMI individuals may experience more discomfort, increasing heart and respiratory rates^31^. Additionally, higher BMI is correlated with reduced structural connectivity within the reward network of the brain^46,47^, potentially influencing motion through disruption of dopaminergic motor circuits, which includes the DMN.

Interestingly, the association between BMI and head motion was consistent within our healthy subject cohort. This suggests that our findings regarding BMI may be applicable to individuals who are metabolically healthy obese (MHO). MHO individuals, while having high BMIs, are not disease-burdened and do not possess the hallmark metabolic impairments associated with obesity^48^. Thus, MHO individuals, who are in good cardiovascular and general physical health as a result of hypertrophy training (i.e., rugby athletes and bodybuilders), would be expected to have higher motion compared to healthy individuals with lower BMI. This notion is supported by Ekhtiari et al. 2019^31^, who observed that BMI alone significantly affected head motion independently of other body composition metrics, such as body fat, water, and lean mass.

In our data, the cardiovascular-related indicators each contributed additional variance to MRI head motion, despite being known to be inter-related^6,49–55^. In our regression analyses, we evaluated the effect of mean arterial pressure on head motion, and in our comparison of disease groups we compared head motion in hypertensives with that of healthy controls. Blood pressure, measured once during a study visit in a research setting, may be less accurate compared to diagnosed hypertension, which is a persistently high blood pressure over time. Blood pressure was measured at the point of consent and not during the imaging visit, which may limit the accuracy of our results. Finally, comorbidity is an important factor in clinical practice which we attempted to control by including the number of ICD10 diagnoses in our analysis, and by repeating our analysis in a group with no recorded lifetime diagnoses. However, we did not differentiate among the types of diagnoses based on likelihood of impacting head motion, which could have limited the usefulness of this measure.

We also identified a significant effect of non-White ethnicity on increased fMRI head motion. While there is a known statistical link between ethnicity and BMI^56,57^, our data did not show this. This may be because the non-white British group comprised a diverse range of ethnicities rather than any single ethnic population (Table S3). Both White and non-White groups had similar means and standard deviations for BMI (White 26.5 ± 4.2, non-White 26.5 ± 4.3). Lastly, the unbalanced sample sizes (non-White: 2.4%, n = 992) may have limited the robustness of this observation.

Mean head motion did not differ significantly across the identified disease categories when compared to healthy controls, except for hypertension. Considering the established relationship between hypertension and the MAP cardiovascular-related indicator, this was expected. Of note, mean motion was higher in the diabetes disease group compared to controls, but not statistically significant despite being a key indicator of head motion in the regression analysis. Several factors may underlie this observation: to ensure unbiased comparisons between disease and control groups, we were limited to disease categories that were of an adequate size. However, this resulted in the inclusion of a broad range of broadly-associated diseases within each disease category group, and may have contributed in diluting the total mean motion observed.

Contrary to previous findings, cognitive task performance resulted in greater head motion, and higher BOLD activation correlated with greater amounts of head motion during task performance. Recent literature has found increased head motion in resting-state fMRI due to susceptibility to arousal confounds and reduced subject compliance^58–60^, although it should be noted that these studies differed greatly from our analyses by including small sample sizes (from 13 to 290) and hard-to-scan populations (i.e. young children) within their paradigms, which may explain the discrepancy in findings.

Unexpectedly, prior MRI scan experience did not reduce head motion; instead, we observed increased motion in both rest and task fMRI conditions during follow-up. However, our findings may be somewhat limited by certain effects, such as the duration between the scans (approximately 5 years) given the elucidation of age as an indicator of motion, alongside the small magnitude of the observed motion difference between baseline and follow-up scans (> 0.01 mm). Familiarity with the MRI scanning paradigm has been theorized to alleviate anxiety and discomfort by diminishing subject claustrophobia and heightened self-awareness^61,62^. Familiarity has also been integrated into other experimental paradigms, especially in hard-to-scan populations^18^. Further research is required to better evaluate the impact of scan experience on head motion.

In agreement with prior research^6,31,34^, the present study has underscored the influence of BMI on MRI head motion. To mitigate the impact of BMI on study outcomes in both healthy and treatment-seeking populations, MRI researchers may opt to tighten their selection criteria by including lower BMI cohorts for study where possible. In analyses that study conditions that make this approach unfeasible, we strongly recommend that researchers invest in active motion reduction strategies, such as acclimation or head moulds^16,17,19^. Future work could include cost–benefit analyses of active motion mitigation versus increased sample size. At some threshold, it is likely more cost efficient to implement active motion mitigation than to further increase sample size. Thus, these studies could weigh theoretic improvements in effect size against cost.

There were several limitations in our study. Our study demographic skewed strongly towards British-White (non-White: 2.4%, n = 992). Our study focused on BMI, where other measures of body adiposity, such as the hip-to-weight ratio, exist. While such measures may be more predictive of mortality and diabetes^63^, BMI was chosen as an indicator due to its widespread use and its more established relationship to fMRI head motion. Secondly, we did not address the effects of motion on other MRI sequences commonly impacted by head motion^64,65^. Our study primarily focused on fMRI motion as it is the most common modality in which motion correction is applied, although other sequences are also affected by motion^66,67^. Thirdly, the UK Biobank cohort studied only included subjects aged 40–69 years, and data was acquired in a research (non-clinical) setting, which may limit the generalizability of our results. On the other hand, the demographic captured in this study presents with the highest engagement of healthcare-seeking behaviour^68^. Our longitudinal analyses did not adjust for changes in other variables over the time interval (e.g. BMI), and our analyses did not account for variances across different imaging centres within the UK Biobank. Lastly, there exists potential for sample bias within the UK Biobank imaging arm due to either incomplete data due to poor health, MRI contraindications, or subject follow-up selection based on lack of incidental findings from baseline^69^.

## Conclusion

This is the largest study of fMRI head motion ranking putative indicators of MRI head motion and evaluating their predictive value. Neuroimaging studies in older or higher-BMI cohorts will benefit the most from motion correction strategies, while the young, low-BMI cohorts will attain the least benefit. Our findings could assist researchers in planning active motion reduction strategies in their image-acquisition protocols. Specifically, motion mitigation is strongly recommended for studies with subjects of higher BMI or older age. Additionally, we provided robust evidence for the impact of post-hoc motion correction of fMRI data, to inform on future study analysis protocols.

## Supplementary Information

Supplementary Tables.

## Data Availability

All individual data used in this study are available by application to the UK Biobank (https://www.ukbiobank.ac.uk/). UK Biobank Field Category Codes for each disease category are available in the supplemental tables.
