# Supplementary material for: Comparison of MRI head motion indicators in 40,969 subjects informs neuroimaging study design
Source: Sci Rep. 2024 Nov 27;14:29430. doi: 10.1038/s41598-024-79827-9 (PMC11603305; doi:10.1038/s41598-024-79827-9)
Supplement: Supplementary file 1 — Supplementary Tables. [file 41598_2024_79827_MOESM1_ESM.docx]

**Table S1.** Disease categories groupings within the UK Biobank population (UK Biobank Data Field 41270) and the number of available datasets containing resting-state fMRI data. Disease categories selected for the final analysis are marked in bold.

| *Disease Category* | *UKB Field 41270 Category Codes* | *Diseases Included* | *N* |
| --- | --- | --- | --- |
| **Psychiatric Disorders** | F00-F99 | Organic mental disorders, Psychoactive mental/behavioural disorders, Schizophrenia, Mood disorders, Neurotic/stress-related and somatoform disorders, Behavioural syndromes associated with physiological disturbances, disorders adult personality, Mental retardation, Disorders of psychological development, Childhood behavioural and emotional disorders, Unspecified mental disorder | **973** |
| **Musculoskeletal Disorders** | M00-M99 | Infectious arthropathies, Inflammatory polyarthropathies, Arthrosis, Other joint disorders, Systemic connective tissue disorders, Deforming dorsopathies, Spondylopathies, Other dorsopathies, Disorders of muscles, Disorders of synovium and tendon, Other soft tissue disorders, Disorders of bone density and structure. Other osteopathies, Chondropathies, Other disorders of the musculoskeletal system and connective tissue | **1096** |
| **Diabetes** | E10-E14 | Diabetes mellitus (Insulin/non-insulin dependent, other specified and non-specified diabetes) | **697** |
| **Hypertension** | I10-I15 | Essential Hypertension, Hyptertensive heart/renal disease, Hypertensive heart AND renal disease, Secondary Hypertension | **1232** |

**Table S2.** One-way ANCOVA comparisons of Estimated Marginal Means for different disease groups. Bolded values denote significant results.

| **Pairwise Comparisons** | | | | | | |
| --- | --- | --- | --- | --- | --- | --- |
| Dependent Variable:  log rest-state fMRI motion | |  |  |  |  |  |
| (I) Table 5 Markers | (J) Table 5 Markers | Mean Difference (I-J) | Std. Error | Sig.^b^ | 95% Confidence Interval for Difference^b^ | |
|  |  |  |  |  | Lower Bound | Upper Bound |
| Control | Psychiatric Disorders | -0.024 | 0.017 | 0.160 | -0.059 | 0.010 |
|  | Musculoskeletal Disorders | -0.005 | 0.012 | 0.678 | -0.029 | 0.019 |
|  | Diabetes | -0.031 | 0.023 | 0.172 | -0.077 | 0.014 |
|  | Hypertension | -.027^*^ | 0.014 | **0.048** | -0.054 | 0.000 |
| Psychiatric Disorders | Control | 0.024 | 0.017 | 0.160 | -0.010 | 0.059 |
|  | Musculoskeletal Disorders | 0.019 | 0.018 | 0.286 | -0.016 | 0.055 |
|  | Diabetes | -0.007 | 0.022 | 0.757 | -0.051 | 0.037 |
|  | Hypertension | -0.003 | 0.017 | 0.864 | -0.036 | 0.030 |
| Musculoskeletal Disorders | Control | 0.005 | 0.012 | 0.678 | -0.019 | 0.029 |
|  | Psychiatric Disorders | -0.019 | 0.018 | 0.286 | -0.055 | 0.016 |
|  | Diabetes | -0.026 | 0.023 | 0.261 | -0.072 | 0.020 |
|  | Hypertension | -0.022 | 0.016 | 0.158 | -0.053 | 0.009 |
| Diabetes | Control | 0.031 | 0.023 | 0.172 | -0.014 | 0.077 |
|  | Psychiatric Disorders | 0.007 | 0.022 | 0.757 | -0.037 | 0.051 |
|  | Musculoskeletal Disorders | 0.026 | 0.023 | 0.261 | -0.020 | 0.072 |
|  | Hypertension | 0.004 | 0.022 | 0.851 | -0.039 | 0.047 |
| Hypertension | Control | .027^*^ | 0.014 | **0.048** | 0.000 | 0.054 |
|  | Psychiatric Disorders | 0.003 | 0.017 | 0.864 | -0.030 | 0.036 |
|  | Musculoskeletal Disorders | 0.022 | 0.016 | 0.158 | -0.009 | 0.053 |
|  | Diabetes | -0.004 | 0.022 | 0.851 | -0.047 | 0.039 |
| Based on estimated marginal means |  |  |  |  |  |  |
| *. The mean difference is significant at the .05 level. |  |  |  |  |  |  |
| b. Adjustment for multiple comparisons: Least Significant Difference (equivalent to no adjustments). |  |  |  |  |  |  |

**Table S3.** Composition of Ethnicity variable. Groupings found within the UK Biobank (Data-Field 21000).

| White British |
| --- |
| Mixed |
| White and Black Caribbean |
| White and Black African |
| White and Asian |
| Any other mixed background |
| Asian or Asian British |
| Indian |
| Pakistani |
| Bangladeshi |
| Any other Asian Background |
| Black or Black British |
| Caribbean |
| African |
| Any other Black Background |
| Chinese |
| Other ethnic group |
